# The Cardioprotective Effects of Polyunsaturated Fatty Acids Depends on the Balance Between Their Anti- and Pro-Oxidative Properties

**DOI:** 10.3390/nu16223937

**Published:** 2024-11-18

**Authors:** Malgorzata Sidorkiewicz

**Affiliations:** Department of Medical Biochemistry, Faculty of Health Sciences, Medical University of Lodz, 90-419 Lodz, Poland; malgorzata.sidorkiewicz@umed.lodz.pl

**Keywords:** PUFAs, LDL-C, atherosclerosis, CVD, atrial fibrillation

## Abstract

Polyunsaturated fatty acids (PUFAs) are not only structural components of membrane phospholipids and energy storage molecules in cells. PUFAs are important factors that regulate various biological functions, including inflammation, oxidation, and immunity. Both *n*-3 and *n*-6 PUFAs from cell membranes can be metabolized into pro-inflammatory and anti-inflammatory metabolites that, in turn, influence cardiovascular health in humans. The role that PUFAs play in organisms depends primarily on their structure, quantity, and the availability of enzymes responsible for their metabolism. *n*-3 PUFAs, such as eicosapentaenoic (EPA) and docosahexaenoic (DHA), are generally known for anti-inflammatory and atheroprotective properties. On the other hand, *n*-6 FAs, such as arachidonic acid (AA), are precursors of lipid mediators that display mostly pro-inflammatory properties and may attenuate the efficacy of *n*-3 by competition for the same enzymes. However, a completely different light on the role of PUFAs was shed due to studies on the influence of PUFAs on new-onset atrial fibrillation. This review analyzes the role of PUFAs and PUFA derivatives in health-related effects, considering both confirmed benefits and newly arising controversies.

## 1. Introduction

Fatty acids (FAs), which are the main components of triglycerides and phospholipids of cell membranes and of reserve fat, are transported in the human body within lipoproteins along with cholesterol and proteins [1]. FAs exert critical effects on health, which include the development of atherosclerosis and cardiovascular risk. Cardiovascular diseases (CVDs) comprise a group of disorders of the heart and blood vessels that are associated with inflammatory processes. Atherosclerosis is a chronic inflammatory disease driven by improper lipoprotein metabolism that, in consequence, causes CVDs [2]. Atherosclerosis starts with the retention of low-density lipoprotein cholesterol (LDL-C) in the subendothelial intima of arteries that causes the activation, followed by dysfunction, of endothelial cells and build-up of fatty deposits inside the arteries [3]. Fatty acids themselves, as well as compounds originating from FAs, are known as mediators of both vascular inflammation and resolution of inflammation [4]. These ambivalent biological functions of fatty acids depend on their quality, quantity, and the availability of enzymes responsible for their metabolism in the human body [5]. The composition of fatty acids varies between individuals of the same species, and additionally, FAs are subject to constant interconversions in each organism. These conversions of FAs depend on both genetic polymorphism in genes coding for enzymes implicated in fatty acids metabolism and on dietary habits. While the former so far remains free from medical manipulations, diet is a matter of individual choice exerting multiple effects on the lipid profile of the body. Firstly, the composition of fats supplied in the diet strongly influences the expression of genes [6]. Secondly, the composition of fatty acids in the diet has a direct impact on the profile of FAs in the body and, in turn, on health. To understand this relationship, one should analyze the structures of FAs and the consequences of their transformations in the body. FAs are carboxylic acids possessing a variable number of carbon atoms that form a hydrocarbon chain terminated by a carboxyl group (on one end) and a methyl group that contains a carbon atom referred to as the ‘n’ or ‘ω’ (on the other end). The chains may vary in length from two to over thirty carbon atoms. The simplest classification divides FAs into the group of saturated fatty acids (SFAs) that have no double bond and the group of unsaturated FAs that possess at least one double bond. Although SFAs are essential for human life [7,8], they have also been identified as a critical risk factor for cardiovascular diseases [9]. Studies have shown that excessive consumption of SFAs leads to an increased concentration of LDL-C [10], known as a causal factor in atherosclerotic CVD [11]. Recently, this simple model is increasingly challenged [12,13]. Only some of the SFAs in the human body are derived from a fat-rich diet; most of them are synthesized de novo as a consequence of an excessive consumption of carbohydrates. Thus, the consumption of SFA only partially influences a harmful elevation of LDL-C [14]. Unsaturated FAs are usually divided into monounsaturated (MUFAs, with only one double bond) and polyunsaturated fatty acids (PUFAs) containing two or more double bonds. According to the position of the first double bond from the omega end (‘n’ or ‘ω’), unsaturated fatty acids can be divided into *n*-3, *n*-6, and *n*-9 groups [15]. It has been demonstrated that a Mediterranean diet enriched in plant MUFAs reduces the risk of CVD [16]. The benefits and safety of PUFAs, especially from a cardiovascular perspective, have also been reviewed in the literature [17,18]. PUFAs are important components of cell membranes, and the changes in relative proportions of PUFAs can influence cell function by the modulation of the fluidity of membranes [19] and by alteration of lipid second messenger synthesis [20]. Unlike SFAs and MUFAs, not all PUFAs can be synthesized in humans due to a lack of necessary enzymes: delta 12 and delta 15 desaturases. Hence, essential PUFAs, linoleic acid (LA, 18:2*n*-6) and alpha-linoleic acid (ALA, 18:3*n*-3), must be provided in the diet as indispensable in of themselves and as substrates for the synthesis of other PUFAs needed for the proper function of the body. Despite frequently indicated benefits of PUFA intake, there is some controversy concerning PUFA’s role as a dietary supplement in certain medical statuses [21,22]. The special interest is focused on the relationship between PUFA supplementation and incidences of atrial fibrillation [23,24]. Thus, the question of the advisability of recommending PUFAs for the general population seems to remain valid [25]. The aim of this review is to summarize what is currently known about the potential benefits of consuming PUFAs, as well as consider the possible side effects that may accompany a diet enriched with PUFA supplements.

## 2. PUFAs Interconversions

**Figure 1:**
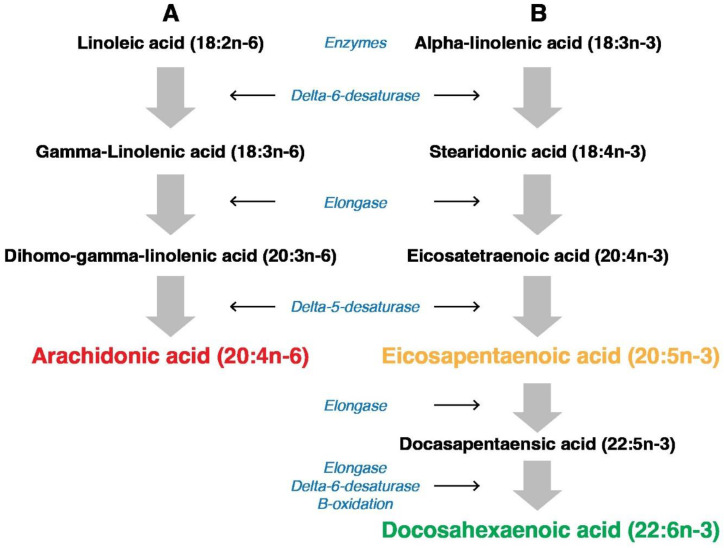
Pathway of conversion (**A**) linoleic acid (LA) into arachidonic acid (AA) and (**B**) alpha-linolenic acid (ALA) into eicosapentaenoic acid (EPA) and docosahexaenoic acid (DHA). As both processes use the same enzymes (delta-6-desaturase, elongase, and delta-5-desaturase), the ratio of LA to ALA influences the ratio between products: AA/EPA + DHA.

Humans can convert fatty acids to longer and more unsaturated ones. The interconversion of PUFAs in humans is possible thanks to the expression of two types of enzymes: elongases and desaturases. But even if fatty acids are converted, newly formed FAs remain in the same group of omega-3, omega-6, or omega-9 acids. This is because new double bonds can be introduced by the enzymatic system in the human body only between the already existing double bond and the carboxyl end of the fatty acid chain (Figure 1). As humans do not express delta 12 and delta 15 desaturases, it is not possible to convert 18C:1*n*-9 acid into 18C:2*n*-6 or 18C:2*n*-6 into 18C:3*n*-3 acid in the human body. Other PUFAs, such as arachidonic acid (AA), eicosapentaenoic acid (EPA), and docosahexaenoic acid (DHA), can be synthesized in the human body from essential FAs, LA and ALA (Figure 1).

It is extremely important as long-chain AA, EPA, and DHA are indispensable components of human tissues, especially lipid membranes, and are incorporated into lipoproteins [26]. Interestingly, the human body’s ability to synthesize each of them is different. AA (20:4*n*-6), a precursor of essential *n*-6 lipid mediators, is both obtained from the diet and synthesized from essential LA [27]. AA is stored in membrane phospholipids and is released by phospholipase A2, mainly from the sn2 position. In turn, phospholipase C is responsible for releasing AA specifically from PI [28]. The second essential FA, ALA, is the potential precursor of important long *n*-3 PUFAs, EPA (20:5*n*-3) and DHA (22:6*n*-3). However, the endogenous synthesis of these main ALA derivatives occurs in the human body to a limited extent [29,30]. Although ALA is the most common omega-3 FA, the efficiency of its conversion in the human body into EPA is usually not sufficient. Similarly, humans have a limited capacity for conversion of EPA into the longer PUFA, DHA. That is why the intake of EPA and DHA from the diet (e.g., oily fish: herring, mackerel, and salmon) or from supplements is recommended as a practical method to maintain a proper concentration of these FAs. In particular, pregnancy increases the demand for long PUFAs. DHA is the most abundant omega-3 PUFA in the mammalian brain, so providing DHA is indispensable for the proper growth and function of the nervous system [31]. The human fetus accumulates DHA in the central nervous system, in particular starting from the second trimester [32]. As was mentioned, mammals obtain DHA from diet, either as DHA itself or as its precursor, ALA. Interestingly, women seem to have a higher level of DHA and a higher capacity for DHA synthesis from ALA compared to men [33]. Concerning dietary intake, it was observed that following supplementation, the concentration of EPA and DHA in cellular phospholipids is elevated, sometimes at the expense of AA [34]. Moreover, the efficacy of omega-3 (ALA) processing into EPA and then into DHA is strongly affected by the concentration of omega-6 (LA) and its conversion into AA. The problem is that both LA and ALA are converted in humans by the same enzyme, delta-6-desaturase. It was observed that the decrease in the ratio between ALA and LA inhibits the conversion of ALA into EPA and DHA [35]. It suggests that competition between PUFAs can be of particular importance for the next steps of PUFAs’ transformation, the formation of lipid mediators in the form of eicosanoids.

## 3. The Role of PUFAs in Eicosanoids’ Formation

**Figure 2:**
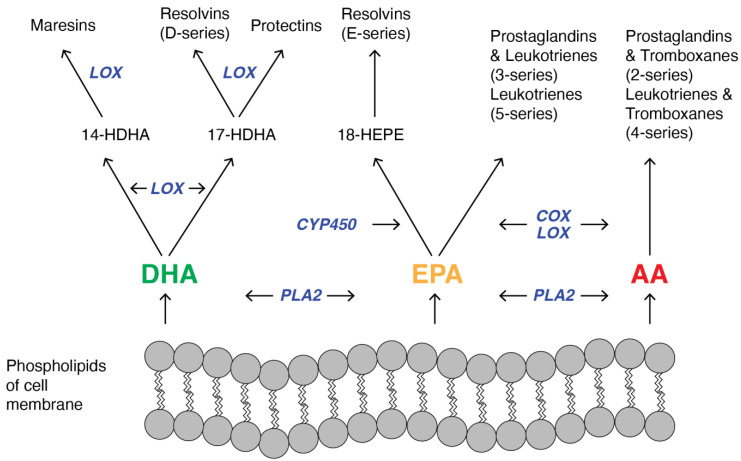
Schematic pathway of synthesis of lipid mediators: maresins, resolvins, protectins, prostaglandins, leukotrienes, thromboxanes from membrane-derived PUFAs: docosahexaenoic acid (DHA), eicosapentaenoic acid (EPA) and arachidonic acid (AA). Substrate competition appears between DHA, EPA, and AA because cyclooxygenase (COX) and lipoxygenase (LOX) catalyze the conversion of both omega-3 and omega-6 PUFAs. In turn, it influences the ratio between the production of anti- and pro-inflammatory mediators. Other used abbreviation: PLA2, phospholipase A2; CYP450, cytochrome P450; HDHA, hydroxyl-DHA; HEPE, Hydroxyeicosapentaenoic acid.

Previously, PUFAs were only considered to be structural components of membrane phospholipids and energy storage molecules in cells. However, it turned out that the main function of PUFAs is to regulate cellular function through the formation of lipid mediators called eicosanoids. On the one hand, due to the role of some eicosanoids in inflammation, they might be implicated in the pathogenesis of atherosclerosis and cardiovascular diseases. On the other hand, the ability of other lipid mediators to resolve inflammation makes them important factors in the prevention of CVD. In particular, AA, EPA, and DHA are precursors of both pro-inflammatory and anti-inflammatory eicosanoids [4]. Eicosanoids comprise different groups of PUFAs-derivatives and include prostaglandins (PG), thromboxanes (TX), leukotrienes (LT), hydroperoxy-(HETE), epoxy-(EET) and specialized pro-resolving mediators: lipoxins (LX), resolvins (Rv), protectins (PD), and maresins (MaR) [36]. They have been recognized as important signaling molecules that are not stored but are synthesized de novo in response to cell activation [37]. Eicosanoids are generated from PUFAs by enzymatic oxygenation pathways involving a distinct family of enzymes: the oxygenases [38]. Precursors and enzymes involved in this synthesis are localized at lipid droplets [37]. The production of the eicosanoids in vivo is influenced by neighboring cells in a process that is described as transcellular biosynthesis [39]. Moreover, intracellular compartmentalization of eicosanoid synthesis seems to be an important factor in the regulation of this process and of the function of eicosanoids. The eicosanoid synthesis (Figure 2) begins with cellular activation that causes an increased influx of calcium and, subsequently, translocation of cytoplasmic phospholipase A2 (PLA2).

PLA2 cleaves PUFA from the sn-2 position of the membrane phospholipid, and this freed fatty acid becomes the substrate for the oxygenase [40]. Different types of oxygenases are involved in the synthesis of lipid mediators (Figure 2), primarily cyclooxygenases (COXs), lipooxygenases (LOXs), and 450 cytochrome epoxygenases (CYP-450). Maresins are synthesized mostly in macrophages from DHA by lipooxygenase via 14-hydroxyl-DHA as an intermediate [41]. Resolvins are derived from both DHA (RvD series) and EPA (RvE series). In the case of RvE biosynthesis, EPA is converted first into 18-hydroperoxyeicosapentaenoic acid by CYP-450, and then this intermediate is metabolized by lipoxygenases and forms RvEs primarily in neutrophils and leucocytes [42]. DHA is not only the substrate needed for the synthesis of resolvins but also protectins. In both cases, DHA is converted into an intermediate that contains 17s-hydroxyl-DHA that is metabolized by lipooxygenases in immune cells [43]. Different series of prostaglandins and leukotrienes are synthesized from eicosapentaenoic acid and arachidonic acid [44], but in the case of both pathways, reactions are catalyzed by LOX and COX. Additionally, thromboxanes are arachidonic acid metabolites [45], and in their synthesis, both COX and LOX participate. As the oxygenation of EPA, DHA, and AA depends on the same enzymes, COXs, LOXs, and CYP-450, it indicates that the quantitative ratio between PUFAs has a significant impact on the type and amount of eicosanoids that are synthesized [35] and in turn, it influences cardiovascular health in humans.

## 4. Cardioprotective Effects of PUFAs

**Figure 3:**
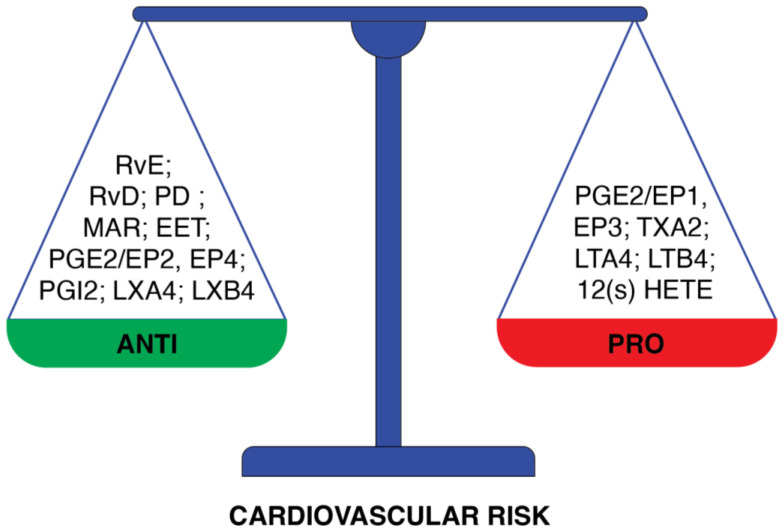
Division of PUFAs-derived lipid mediators according to the cardiovascular risk that they present. The group of mediators of anti-cardiovascular risk properties comprises: resolvin E (RvE), resolvin D (RvD), protectin (PD), maresin (MaR), 14-15 epoxyeicosatrienoic acid (EET); prostaglandin E2 through EP2 and EP4 prostaglandins receptors (PGE2/EP2, EP4), prostacyclin 2 (PGI2), lipoxin A4 (LXA4) and lipoxin B4(LXB4). Opposite properties are presented by: prostaglandin E2 through EP1 and EP3 prostaglandins receptors (PGE2/EP1, EP3), thromboxane A2 (TXA2), leukotriene A4 (LTA4), leukotriene B4 (LTB4), 12-hydroxyeicosatetraenoic acid (12(S)-HETE).

Cardiovascular diseases due to atherosclerosis remain the main cause of death worldwide [46]. Thus, hypercholesterolemia, which contributes to the development of atherosclerosis, is a prominent cardiovascular risk factor. Although LDL-C is ubiquitous and irreplaceable in the body, increased LDL concentration and accumulation have a considerable effect on the risk of coronary diseases [47]. For this reason, it is assumed that LDL-lowering therapy is the most important anti-inflammatory approach to prevent CVD [48]. As to the role of PUFAs in the context of cardiovascular risk, the ability of PUFAs to decrease plasma LDL-C concentration by increasing the expression of LDL receptors seems to be critical [10]. Marine fishes, an important source of PUFAs, particularly those from the omega-3 group, have beneficial effects and therapeutic potential in chronic inflammatory diseases. The discovery of *n*-3 PUFAs as dietary interventions for inflammatory diseases originated from epidemiological studies conducted on populations of Greenland Inuit, Alaskans, and Okinawa Japanese consuming a traditional marine diet [49,50,51,52]. From these first published observations, many studies confirmed an association between high omega-3 intake and low cardiovascular risk and reduced CVD morbidity or mortality. It was observed that the dietary intake of omega-3 PUFAs decreases the concentration of circulating triglycerides, which in turn reduces the synthesis of an LDL precursor, VLDL [53,54]. It seems important since, in the case of hepatic overproduction of VLDL, the accumulation of triglyceride-rich lipoprotein remnants in the blood is observed. Furthermore, it was shown recently that elevated remnant triglycerides are significantly associated with higher cardiovascular risk [55]. A protective effect against cardiovascular diseases was observed in the case of studied people on the Mediterranean diet enriched in omega-3 PUFAs [56]. Altered membrane fluidity and decreased activity of HMG-CoA reductase, responsible for LDL synthesis, are other effects observed after increased consumption of omega-3 PUFAs-rich fish oil [57]. The biophysical membrane analysis [19] revealed that the presence of PUFAs in membranes increases not only membrane fluidity but also elasticity and flexibility. Similar results were found in a multi-ethnic cohort study [58], where the levels of circulating omega-3 and omega-6 FAs were measured. Higher plasma levels of omega-3 PUFAs were associated with an increased arterial elasticity, which in turn reduced the risk of atherosclerosis. It was demonstrated that an EPA- and DHA-treated group of adults presented a reduced level of circulating pro-inflammatory cytokines in comparison to a control group [59]. Omega-3 PUFA supplementation, in another clinical study, also demonstrated a positive effect on reducing the incidence of cardiovascular death and myocardial infarction [60]. As the increase in dietary PUFA intake translates into an increased content of PUFAs in cell membranes, the measurement of fatty acid incorporation as phospholipids in red blood cells (RBC) membranes became a commonly used method to assess this intake [61,62,63]. An analysis of the RBC FA profile is followed by a determination of the omega-3 index, the percentage of EPA plus DHA in erythrocyte membrane in comparison to other FAs. The study on RBC membrane assessed on type 2 diabetes patients [64] showed the strict correlation between membrane FAs composition and membrane fluidity quantified as general polarization. This lipidomic analysis suggests that the higher content of arachidonic acid and increased *n*-6/*n*-3 ratio were related to the lower general polarization. Proportions of EPA plus DHA in erythrocyte phospholipids closely correlate with the content of these fatty acids in cardiac tissue [65]. Moreover, it was shown that the structural properties of highly unsaturated DHA/EPA-rich phospholipids contribute essential fluidity to the bilayer and support a respiratory lifestyle dependent on proton bioenergetics [26]. The omega-3 index was found to be an independent predictor of CVD risk [66]. On the other hand, the *n*-6 PUFA effect on the cardiovascular system is not so clear. In the case of linoleic acid (LA), which is the main source of *n*-6 FAs in our diet, the positive effect of this FA was observed in decreasing blood pressure and inflammation, but it is not observed for arachidonic acid (AA). [67,68]. The role of lipid mediators in the resolution of inflammation (Figure 3) depends on the ratio between PUFA substrates (AA, EPA, DHA) used for the synthesis of mediators. Anti-inflammatory lipid mediators are derived mostly from omega-3 PUFAs and include E-series resolvins (RvEs) that are synthesized from EPA, and D-series resolvins (RvDs), protectins (PDs), and MaRs that are formed from DHA [36,69]. However, omega-6 PUFA derivatives show anti-cardiovascular properties.

Lipoxins (LXA4, LXB4) that are synthesized from arachidonic acid in mucosal tissues, in blood vessels, and in platelets induce a biochemical transition from inflammation to resolution [70]. Epoxyeicosatrienoic acid (EET), another mediator derived from AA, plays an important role in cardiovascular protection due to its anti-inflammatory properties [71]. On the other hand, many other mediators derived from AA, like leukotrienes (LTs), thromboxanes (TXs), and prostaglandins (PGs), show properties that increase cardiovascular risk. Leukotrienes increase CVD risk as a potent coronary artery vasoconstrictor [72]. Thromboxanes, vasoconstrictors related to platelet aggregation, are involved in atherosclerosis development [45]. Another lipid mediator, 12-hydroxyeicosatetraenoic acid (12(S)-HETE) derived from AA, is known as a pro-inflammatory chemoattractant for neutrophils [73]. Interestingly, the function of prostaglandins depends on their series and type of receptor. EP1 and EP3 receptors are responsible for blood vessel constriction. In case of blood vessel dilation, EP2 and EP4 involvement is necessary [74,75]. PGE2 inhibits the production of inflammatory leukotrienes and induces the production of inflammation-resolving lipoxin A4 [76]. It was demonstrated that endothelial cells treated with EPA produce PGD3, which antagonizes the neutrophil PGD2 receptor, inhibiting neutrophil migration [77].

**Figure 4:**
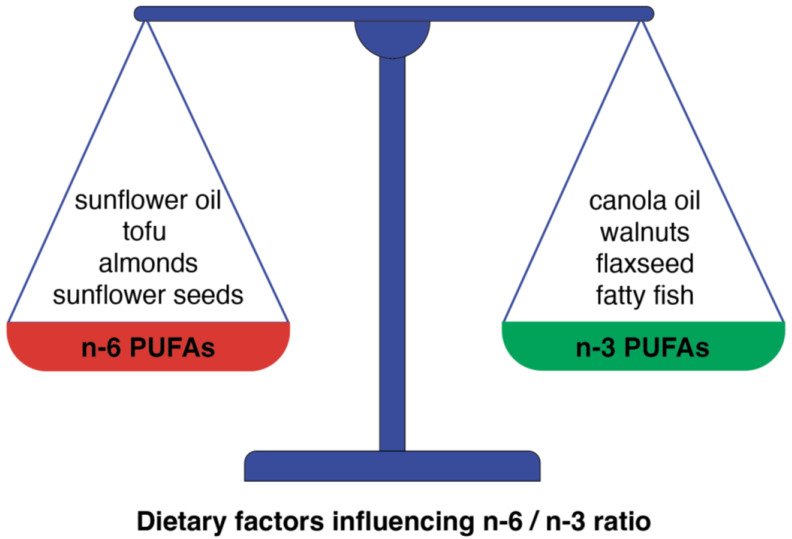
The dietary-dependent balance between *n*-6 and *n*-3 PUFAs is a crucial factor for the prevention of CVD. This scheme presents some examples of food products in which the predominance of *n*-6 PUFAs can be found and products in which *n*-3 PUFAs predominate.

As lipid mediators originating from PUFAs display both anti-and pro-inflammatory properties (Figure 3), the imbalance between their formation can tip the scales toward the development of cardiovascular disease [78]. Generally, decreasing the ratio of *n*-6 to *n*-3 PUFAs can shift the balance of eicosanoid production to a less inflammatory profile and could be beneficial in the context of vascular inflammation and atherosclerotic events. The level of *n*-6 and *n*-3 PUFAs in the human body is determined by both endogenous metabolism and dietary intake. Thus, the need for a balanced dietary intake of these PUFAs seems to be essential for metabolic health (Figure 4).

Whereas an *n*-6/*n*-3 ratio of 5:1 could prevent CVD, an increased intake of *n*-6 PUFAs, currently observed in the Western diet, resulting in a four times higher *n*-6/*n*-3 ratio, enhances CVD incidences [79] through the activation of the inflammatory pathways. The competition between *n*-6 and *n*-3 FAs for the same enzymes at different stages of metabolism of PUFAs (Figure 1 and Figure 2) in combination with the observed increase in the intake of *n*-6 PUFAs ultimately results in inflammation and, in turn, CVD incidences. And vice versa, an increased *n*-3 PUFA intake, due to its anti-inflammatory properties, seems to be beneficial for cardiovascular health. However, despite all the promising data mentioned above, some effects of PUFAs, especially on the incidence of atrial fibrillation, remain controversial.

## 5. PUFAs and Atrial Fibrillation

Atrial fibrillation (AF) is now the most prevalent cardiac arrhythmia globally and has developed recently into a cardiovascular epidemic [80]. AF is linked with an increased risk of cardiovascular complications, like stroke, that is believed to be associated with AF-dependent hypercoagulation. Mostly because of the increased risk of cardiovascular and renal diseases, patients with AF represent a group at high risk of mortality [81]. Although the pathogenesis of AF looks complicated, factors like inflammation, atrial structural remodeling, and dysregulation of the metabolism of fatty acids seem to be predominant contributors to this arrhythmia. Concerning inflammation, omega-3 PUFAs, thanks to their anti-inflammatory and anti-fibrotic effects, have been suggested to reduce the incidence of AF [82,83,84]. In contrast, *n*-6 PUFA-derived eicosanoids have been assumed to be pro-inflammatory [76]. The imperfection of this simple scheme can probably explain the controversial results obtained in the studies on the effects of PUFAs on AF incidents. In a population-based cohort study [85], an increased dietary intake, as well as a higher circulating concentration of omega-6 PUFAs, especially linoleic acid (LA), were associated with a lower risk of atrial fibrillation. A similar inverse association between a lower risk of AF and the plasma concentration of *n*-6 PUFAs was found in an earlier study [86]. Another multi-ethnic cohort study also demonstrated that plasma concentration of *n*-6 PUFAs (in this case, particularly AA) was associated with a lower risk of the incidence of AF [87]. The study conducted among older adults demonstrated an association between the serum level of non-esterified FA and new-onset atrial fibrillation. Serum level of nervonic acid (24:1, *n*-9) was positive, whereas gamma-linolenic acid (18:3, *n*-6) was inversely associated with the incidence of AF [88]. However, potentially anti-inflammatory PUFA3 has been shown to be a factor in promoting atrial fibrillation in many studies. The study of changes in EPA and DHA serum concentration during long-term PUFA supplementation showed a significant correlation between EPA serum level and a higher risk of AF and a similar tendency for DHA [89]. In the case of elderly patients after myocardial infarction, omega-3 PUFA supplementation increased the risk of atrial fibrillation [90]. A dose-related increase in AF risk as a result of omega-3 PUFA supplementation (in the range of 1-4 g/d) was found in randomized controlled trials [91]. A meta-analysis of AF risk in patients supplemented with icosapent ethyl (purified version of EPA) demonstrated an increased risk of this type of arrhythmia. A larger percentage of patients in the icosapent ethyl group than in the placebo group were hospitalized for atrial fibrillation [92]. A different meta-analysis demonstrated that omega-3 PUFA supplementation increased the risk of AF, and a significant dose–response was observed in new-onset AF with EPA plus DHA supplementation [25]. To find an explanation for these controversial results, one should analyze the mechanism of atrial fibrillation. One of the important AF indicators is sustained atrial dilatation, which indicates the role of mechanical overload in the pathophysiology of AF. It suggests that atrial fibroblasts react to mechanical forces due to the presence of a Piezo1 ion channel that is responsible for mechanical stress-induced signaling in fibroblasts [93]. The fact that the increased Piezo1 activity was found in patients with AF suggests the role of this channel in the structural and electrical remodeling of the atrium [94]. Interestingly, it was observed that omega-3 PUFAs can affect Piezo1 channel activity [95] by prolonging the action potential duration that triggers AF and the delayed polarization [93]. In turn, fibrosis seems to be an important factor in the process of atrial structural remodeling in AF development. A study comparing plasma from AF patients and normal human plasma showed an elevation of biomarkers of collagen remodeling and fatty acids’ metabolism [96]. These findings suggest that both collagen remodeling and fatty acid dysregulation are implicated in AF pathogenesis. An analysis of the modulation of these markers can provide additional information about the pathophysiology of atrial remodeling.

## 6. Conclusions

In conclusion, despite many trials investigating the cardioprotective effects of polyunsaturated fatty acids, the results remain confusing. Although *n*-3 PUFA dietary intake/supplementation seems to be an effective strategy to prevent cardiovascular outcomes, the association of *n*-3 intake with AF risk suggests that special caution be exercised when high doses are used. In turn, *n*-6 PUFAs, despite their known role in the synthesis of pro-inflammatory mediators, have shown potent anti-fibrillatory effects.
